# HIV, HBV, HCV and *T. pallidum* infections among blood donors and Transfusion-related complications among recipients at the Laquintinie hospital in Douala, Cameroon

**DOI:** 10.1186/2052-1839-14-5

**Published:** 2014-02-12

**Authors:** Carole Else Eboumbou Moukoko, Françoise Ngo Sack, Estelle Géraldine Essangui Same, Madeleine Mbangue, Léopold Gustave Lehman

**Affiliations:** 1 Department of Biological Sciences, Faculty of medicine and pharmaceutical sciences, University of Douala, BP 2701 Douala, Cameroon; 2 Pole d’Excellence en Epidémiologie du Paludisme, Centre Pasteur du Cameroun, Yaoundé, Cameroon; 3 Department of Hematology and Medical Oncology, Yaoundé Central Hospital, Yaoundé, Cameroon; 4 Laboratory of Physiology and Animal Biology, Faculty of science, University of Douala, Douala, Cameroon; 5 Blood Transfusion Center, Laquinitinie Hospital, Douala, Cameroon

**Keywords:** Blood transfusion, HIV, HBV, HCV, *T. pallidum*, Complications

## Abstract

**Background:**

Transfusion-transmissible infections (TTIs) pose a major health risk in Cameroon given the high prevalence of such pathogens and increased demands for blood donations in the local communities. This study aims at establishing the prevalence of commonly encountered TTIs among blood donors and transfusion-related complications among recipients in an urban center of Cameroon.

**Methods:**

A total of 477 blood donors and 83 blood recipients were recruited by consecutive sampling at the Laquintinie Hospital in Douala (LHD), Cameroon. Serum samples from blood donors were tested by quantitative enzyme-linked immunosorbent assays (ELISA) and/or using various Rapid diagnostic test (RDT) for presence of Hepatits B (HBV) viral antigens, and antibodies to human immunodeficiency (HIV-1/2), Hepatits B (HCV) and *Treponema pallidum*. Recipient’s medical records were also analyzed for possible transfusion-associated complications.

**Results:**

The male/female sex ratio of the blood donors was 4/1 with a mean age of 30.2 (Sd = 8.3) years. Of all blood donors, 64/467 (13.7%) were infected by at least one of the four TTIs. Infected volunteer donors represented 8.3% while infected family donors comprised 14.3% of the donor population. The prevalence of HCV, HIV, HBV and *T. pallidum* were 1.3%, 1.8%, 3.5%, and 8.1%, respectively. More than half of the blood recipients were female (78.3%) and the mean age was 20.6 (SD = 16.1) years. The causes of severe anemia indicative of transfusion in recipients varied with wards (postpartum hemorrhage, caesarean section, uterine or cervical lacerations, abortions, urinary tract infections, severe malaria, vaso-occlusive attacks, wounds and gastrointestinal bleeding). The most frequent complications were chills and hematuria, which represented 46.1% of all observed complications. Other complications such as nausea, vomiting, jaundice, sudden diarrhea, anxiety, tachycardia, or hyperthermia were also found in recipients. Three cases of deaths occurred during the study, including a girl of less than one year.

**Conclusion:**

This study confirms the presence of blood-borne infectious diseases in blood donors at the LHD, identifying *T. pallidum* as the greatest threat to blood safety in the region, and hematuria as the most common immunological complications in blood recipients.

## Background

Blood transfusion therapy is used among patients with severe anemia due to various medical, surgical or obstetric conditions, and in patients undergoing transplantation of an organ. Blood transfusion is beneficial and safe for the recipient when it is performed in strict compliance with immunological and hygienic standards, and following a strict screening of donors. In Cameroon, the current blood safety guidelines necessitate blood banks to routinely perform serological testing for human immunodeficiency virus (HIV), hepatitis B virus (HBV), hepatitis C virus (HCV), and *Treponema pallidum* (*T. pallidum*). These guidelines progressively followed the exponential rise in blood donors from 75,000 in 1992 to 130,000 in 2002 [1], however, necessitate regular monitoring and adaptation to frequently changing epidemiological and demographic parameters that include urbanization, migration flows, and increased demand for blood transfusion in the country. Although blood safety has greatly improved over the past 15 years, TTIs still represent a major public health problem in Cameroon given the high prevalence of HIV infections, hepatitis, malaria, and several sexually transmissible diseases (STD) [2,3]. Data recorded in 2006 show that 26,079 units of blood were collected in health facilities in Cameroon with over 2,477 infected cases, thus a TTIs prevalence of 9.5% among blood donors. Of these infectious risks, viral infections (HIV, HBV and HCV) are the most feared by patients and prescribers [4-7]. In Cameroon, screening for hepatitis B and C virus was not part of routine tests performed in blood donors until the year 2005. The reduction of the residual risk of contamination is currently based on a strict selection of donors and the introduction of new tests such as genomic testing for HIV, HCV and HBV [6,8]. A study conducted among blood donors at the Yaoundé Central Hospital (YCH) revealed that the risk of TTI from patients with residual infections remains high (9.8%) in Cameroon [9]. Bacterial contamination remains a major risk of infection during blood transfusions. Endotoxic shock caused by massive, usually Gram-negative, bacterial contamination is rare but represent a very serious outcome that includes sudden death [10]. Bacterial infections including *T. pallidum* have been reported in Cameroon, and are common in many other countries [11-14].

Despite the considerable efforts in limiting infection-related complications of blood transfusion, the risk of adverse pleiotropic reactions to transfused blood remains present. Among these transfusion-related complications, immunological reactions and hypervolemia are the most common [10,15]. Some reactions happen as soon as the transfusion is started, while others take several days or months to develop. Evaluation of transfusion complications in blood recipients is necessary for a full understanding of its etiology, and must involve a permanent and systematic collection and reporting of cases at blood transfusion centers. In 2003, a law on the rapid and proper cases management at local health facilities free of charge has been adopted in Cameroon to complete institutional reforms on blood safety [16].

Till date, very few studies have been carried out in Cameroon to assess the complications due to transfusion and most of them concern the prevalence of infections in blood donors [9,16,17]. This study aimed to study the prevalence of common TTIs among blood donors and to analyze the possible complications due to blood transfusion among recipients at the Laquintinie Hospital in Douala (LHD), a major blood transfusion center in the coastal region of Cameroon.

## Methods

### Study population and design study

We conducted a four-month transversal descriptive survey starting from August 2012 at LHD. We included individuals attending the blood transfusion center (BTC) for voluntary or family blood donation, and hospitalized patients in the Pediatric, Emergency, and Gyneco-obstetrical wards who recently underwent blood transfusion. Before enrollment, subjects or patients, or parents, or legal guardians were informed on the purpose and process of the investigation (goals, methodology, study constraints, data confidentiality, and rights to opt out from the study), and a written informed consent was obtained from all participants. Standard questionnaires and data collection sheets were used to collect anthropometric, socio-demographic, and clinical and biological data. This study was conducted in accordance with ethics directives related to research on humans in Cameroon. Ethical clearance was obtained from the LHD institutional review board, whereas administrative clearance was obtained from the Regional Delegation of Public Health for the Littoral Region.

### Detection of viral and bacterial infections

**Figure 1:**
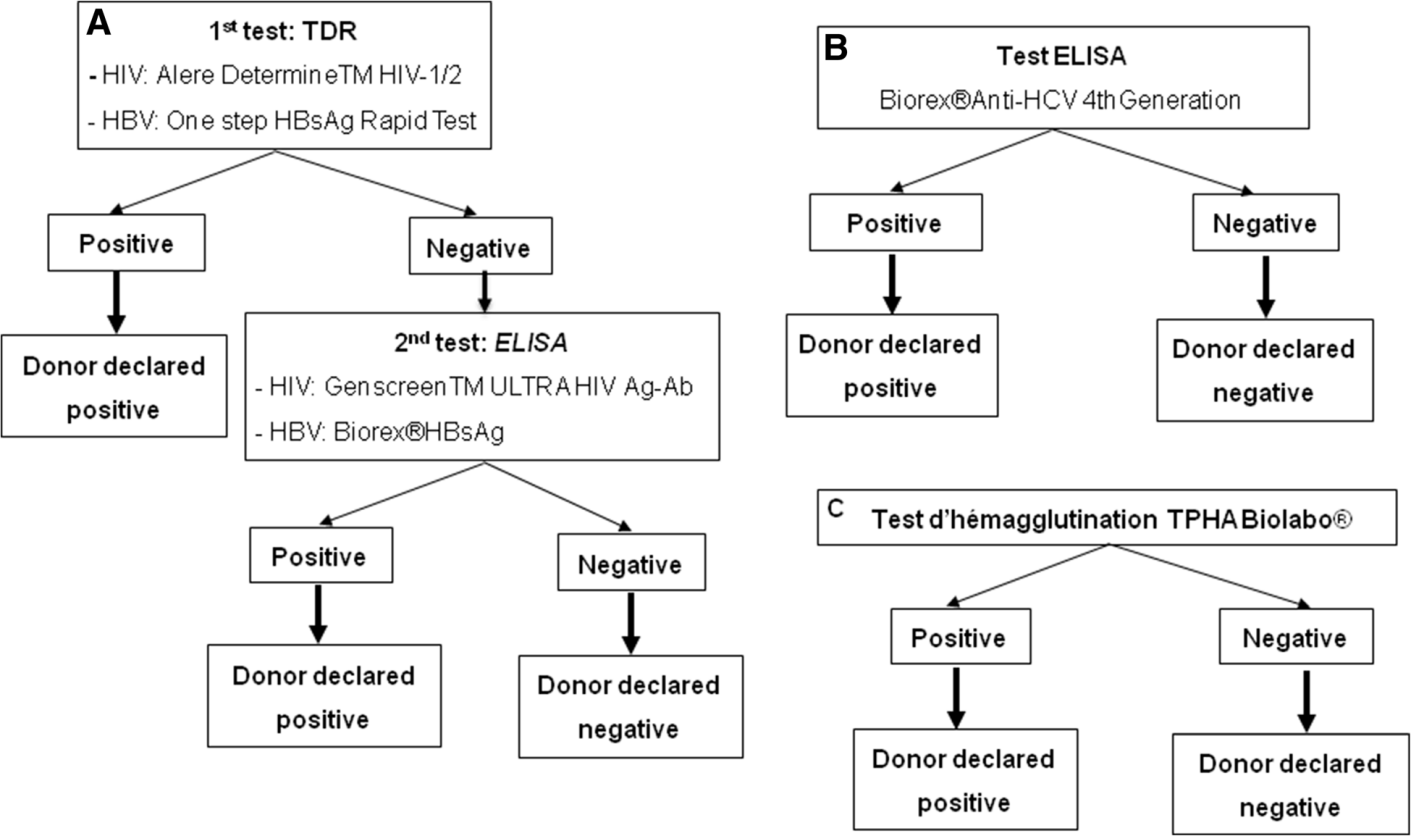
**Viral and bacterial infections testing strategies and *algorithms. *A**, HIV and HBV diagnostic; **B**, HCV diagnostic; **C**, *T. Pallidum* diagnostic.

For each donor, 10 ml of whole blood were collected in a dry tube. After centrifugation at 3000 rpm for 5 min, serum was collected for the detection of viral and bacterial infections as depicted in Figure 1. A rapid diagnostic test (RDT) (Alere Determine™ HIV-1/2) was used for the qualitative detection of anti-HIV-1/2 antibodies, whereas an enzyme-linked immunosorbent assay (ELISA Genscreen™ ULTRA HIV Ag-Ab) was employed to detect the viral antigens in the plasma of donors. For HBV, two tests were also used; a RDT-based assay (One Step HBsAg Rapid Test) for the qualitative detection of HBV surface antigen (HBsAg), and an ELISA test (HBsAg Biorex®) for quanlitative detection of HBsAg. An ELISA test (Biorex 4^th^ Generation® Anti-HCV) was also used for the detection of anti-HCV antibodies in the plasma and the *T. pallidum* hemagglutination (TPHA) test (Biolabo®) was used for the qualitative and semi-quantitative detection of anti-*T. pallidum* antibodies.

### Blood group determination, cross matching and transfusion-related complications

Samples were typed for ABO blood groups, using the "Beth Vincent" and Simonin-Michon methods. Blood unit samples were also cross-matched to confirm that they matched the blood type of the recipient. Transfusion reactions were collected from recipient patients’ medical records.

### Statistical analyzes of data

Data were presented as either grouped frequency tables, or as means ± Standard Deviations (SDs) for normally distributed numerical variables. Chi-square test or Fisher’exact test were used for comparing proportions. Numerical values were compared using the U-test of Wilcoxon-Mann–Whitney. All statistical analysis were done using the Excel and Stata software package (version 11 SE). Only *p-values <0.*05 were considered significant in these analyses.

## Results

### Blood donors’ population study

#### Characteristics of blood donors

**Figure 2:**
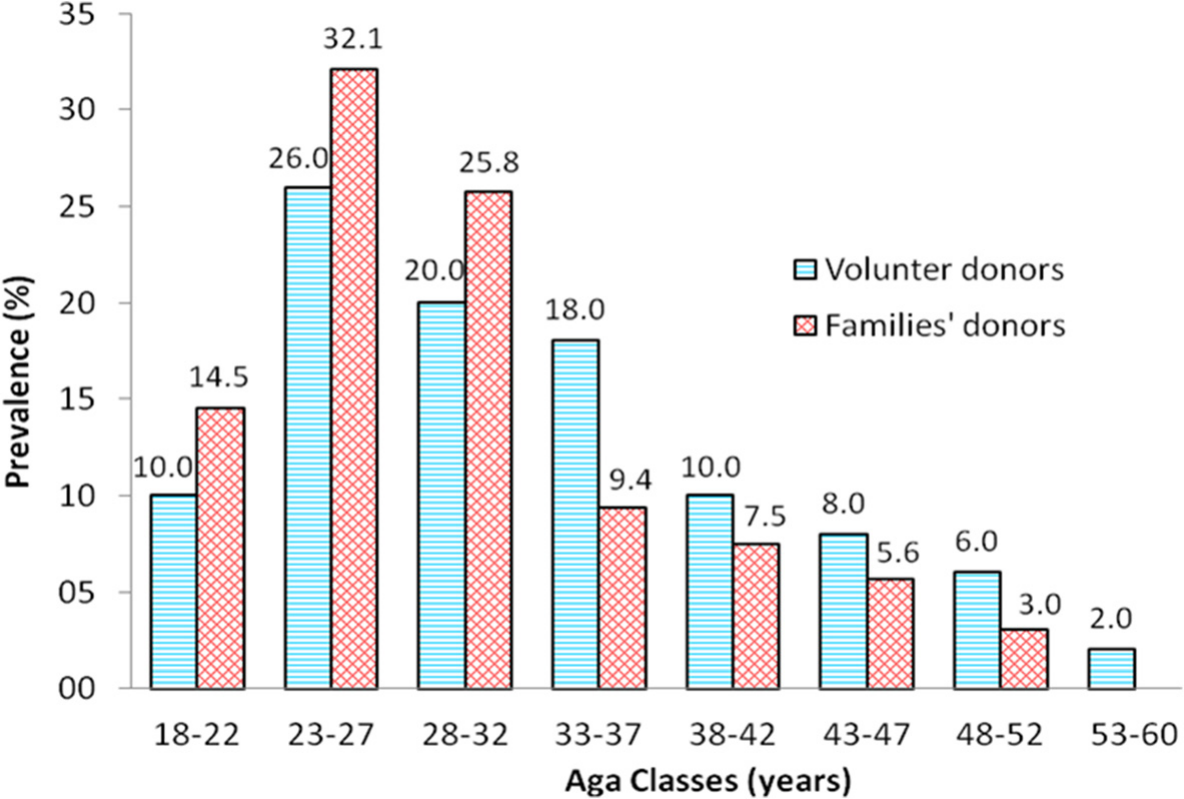
Distribution of blood donors’ groups according to age classes.

We included 477 blood donors in the study, 50 (10.5%) were volunteer donors whereas 427 (89.5%) were family donors. Overall, the male/female sex ratio amongst all donors was 4/1 (381/96). The demographic characteristics of both the volunteer and family donors are presented in Table 1. We observed a significantly higher mean age of the volunteer donors compared to that of the family donors (p =0.0017). No difference was observed for all other identified characteristics of the participants. Blood donors were more frequent in the 23–27 years age group, and decreased thereafter (Figure 2). The majority of donors of age < 32 years were family members of the recipients, whereas volunteer donors dominated the >32 years age groups.

**Table 1:** Sex, age, anthropometric and clinical characteristics of blood donors

	**Volunteer donors**			**Family donors**				
	**Male**	**Female**	**Total**	**Male**	**Female**	**Total**	**Total**	**p**
	**(N = 37)**	**(N = 13)**	**(N = 50)**	**(N = 344)**	**(N = 83)**	**(N = 427)**	**(N = 477)**	
Age (SD), years	37.7 (9.6)	27.8 (5.2)	32.9 (9.2)	29.4 (7.9)	31.6 (8.7)	29.9 (8.2)	30,2 (8.3)	0.017
Weight (SD), Kg	77.6 (12.4)	66.9 (5.9)	74.8 (11.9)	75.5 (11.6)	70.3 (13.3)	74.5 (12.1)	74.5 (12.1)	0.94
Body mass index (SD), Kg/m^2^	25.5 (3.5)	26.1 (2.7)	25.7 (3.3)	24.9 (3.7)	26.3 (5.2)	25.3 (4.0)	25.3 (3.9)	0.11
Systolic blood pressure (SD), mmHg	131.2 (14.5)	113.1 (11.4)	126.4 (15.8)	129.9 (16.2)	120.2 (15.2)	128.0 (16.4)	127.8 (16.3)	0.47
Diastolic blood pressure (SD), mmHg	76.8 (9.8)	75.0 (8.8)	76.3 (9.5)	77.0 (11.2)	77.0 (10.6)	77.0 (11.0)	76.9 (10.9)	0.87
Pools (SD), bpm	76.6 (88.2)	78.7 (4.2)	77.2 (7.4)	77.3 (12.7)	83.1 (11.7)	78.4 (12.7)	78.3 (12.3)	0.62

Data are mean (SD) of various parameters according to donors' groups and gender; Variables were compared by Cross Kwalis test (continuous variables) and p < 0.05 was significant.

Most of donors (60.3%) had received secondary-school education, 31.2% attended university studies, and 6.6% had completed primary school. This distribution is significantly different (p <10^-4^) between the two donors’ groups. In volunteer donors, 81.8% attended university studies and 18.2% had received secondary-school education. 64.0% of family donors had received secondary-school education, 26.80% attended university studies and 7.2% had completed primary school.

Most of donors (52.2%) belong to the O blood group whereas 25.6%, 18.7% and 3.5% belong respectively to the A, B and AB blood groups. About 63% of these donors were Rhesus positive.

Data on transfusion history were collected from 209 (209/427) family donors and 9 (9/50) volunteer donors. Among these, 1.4% of family donors had previously received a transfusion compared to 11.1% of the volunteer donors. 66% of the volunteer donors had previously donated blood compare to 48.3% of the family donors (p = 0.03).

#### Seroprevalence of TTIs among blood donors

Eight donors (1.77%) were positive for anti-HIV antibodies and were all family donors (Table 2). Out of 461 donors tested for HBsAg, 16 (3.5%) were positive, the majority (93.7%) being family donors. Six donors (1.3%) were positive for anti-HCV antibodies and 35 (8.1%) for anti-*T. pallidum* antibodies. Among blood donors with at least one infection, most of them (85.9%) were males. Among the donors, only one had a double infection (HCV and *T. pallidum*). None of the infected donors had previously received blood transfusion. No rcorrelation was found between the presence of TTIs and other transmission risk factors such as body piercing, tattoo, sexual promiscuity, prostitution, drug abuse, or blood-related accidents.

**Table 2:** Seroprevalence of infections risk in blood donation

	**Volunteer donors**				**Family donors**				**Total**
	N^*^	**Male**	**Female**	**Total**	N^*^	**Male**	**Female**	**Total**	
VIH (%)	47	0 (0.0)	0 (0.0)	0 (0.0)	406	7 (87.5)	1 (12.5)	8 (1.9)	8 (1.8)
VHB (%)	48	1 (100.0)	0 (0.0)	1 (2.1)	413	13 (86.7)	2 (13.3)	15 (3.6)	16 (3.5)
VHC (%)	47	1 (50.0)	1 (50.0)	2 (4.3)	396	4 (100.0)	0 (0.0)	4 (1.0)	6 (1.3)
*T. Pallidum* (%)	47	1 (100.0)	0 (0.0)	1 (2.1)	383	29 (85.3)	5(14.7)	34 (8.9)	35 (8.1)

Data are frequency (%) of various infections according to donors' groups and gender; *, Total number of donors where the diagnostic test was interpreted.

### Blood recipients’ population study

#### Characteristics of blood recipients

**Figure 3:**
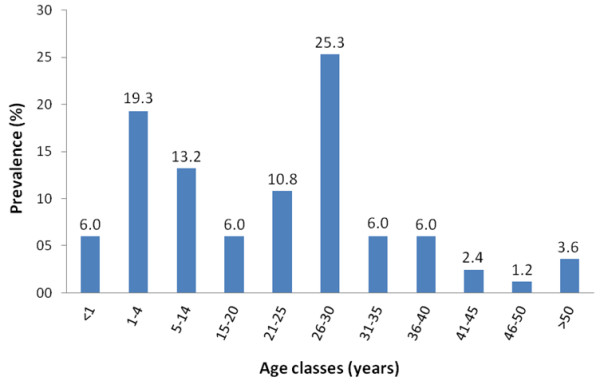
Distribution of blood recipients according to age classes.

The frequency and proportion of blood recipients according to the ward, sex, hemoglobin concentration and mean duration of transfusion are shown in Table 3. A total of 83 transfused patients agreed to participate in the study. More than half of those who received transfusion were female with a female/male sex ratio of 3.6/1. The mean age of recipients was 20.57 (range 0–86 years, SD = 16.09) years, with two notable high frequency age groups: 1–4 years olds and 26–30 years olds (Figure 3). The mean duration of transfusion was higher in the Pediatric ward than in the Gyneco-obstetrical ward, consistent with the guidelines for transfusion in children. The number of recipients who had received at least one blood transfusion prior to the current visit was 26 (31.3%) (Table 3). In the Pediatric ward, 7 (58.3%) of the 12 recipients who had received at least one previous blood transfusion were girls. In the emergency ward, the 2 recipients who had received at least one previous blood transfusion were men.

**Table 3:** Characteristics of blood recipients according to wards

	**Wards**			
	**Gyneco-obstetrical**	**Pediatric**	**Emergency**	**Total**
	**(N = 43)**	**(N = 36)**	**(N = 4)**	**(N = 83)**
Male (%)	0 (0.0)	16 (44.4)	2 (50.0)	18 (21.7)
Female (%)	43 (100.0)	20 (55.6)	2 (50.0)	65 (78.3)
Mean age (SD), years	29.1 (7.8)	6.8 (8.7)	53.2 (24.5)	20.6 (16.1)
Mean haemoglobin^$^ (SD), g/dL	5.6 (1.9)	6.5 (3.4)	2.8	6.0 (2.9)
Mean duration of transfusion (SD), min	191.9 (104.9)	254.6 (167.7)	200.0 (24.5)	220.2 (137.1)
Previous blood transfusion (%)	12 (27.9)	12 (33.3)	2 (50.0)	26 (31.3)

Data are frequencies (%) or mean (SD) according to wards; ^$^Information on 47 individuals (19, 27 and 1 respectively in Gyneco-obstetrical, Pediatric and Emergency wards).

#### Transfusion indications

All recipients in the study were transfused for severe anemia. The causes of anemia varied from one ward to the other. In the Gyneco-obstetrical ward, the main causes of anemia were hemorrhagic (postpartum haemorrhage, caesarean section due to placenta previa, cervical damage, or voluntary abortions) and/or due to urinary tract infections. In the Pediatric ward, anemia was mostly due to malaria (severe malaria and recurrent malaria). Hemolytic causes such as vaso-occlusive crises were the leading cause of transfusion in sickle cell patients in the Pediatric ward. In the Emergency ward, severe hemorrhagic anemia was noted in one patient with knife injuries and another with a digestive haemorrhage.

#### Complications recorded

From clinical records of transfused patients, we reported 10 different transfusionnal complications (Table 4). Overall, 26 (31.3%) recipients presented with transfusion-related complications. Among these, chills and hematuria accounted for 46.1% (12/26), the majority (57.7%) of which were recorded in the Gyneco-obstetrical ward. 34.6% (9/26) and 7.7% (2/26) of the complications were recorded at the Pediatrics and Emergency wards, respectively. A significant trend was observed between multiple transfusions and occurrence of complications (p = 0.05). Three deaths were recorded during the study. The first patient was a 27 years old male with sickle cell disease; he died two days after the transfusion and had previously received one blood transfusion. His clinical record indicated severe anemia (hemoglobin level 3.8 g/dl), and joints and lumbar pains. The second was a 2 years old female who died seven days after admission. Her clinical record indicated severe anemia, palour, hematuria, nausea and vomiting. She died following a sudden cardiac arrest before receiving the second blood unit following a shortage of blood group O in the hospital blood bank. The third patient, a girl under one year old, died two days after transfusion. Her clinical record also indicated anemia (hemoglobin level = 10.3 g/dl) and hyperthermia.

**Table 4:** Transfusion complications in blood recipients according to wards

**Wards**				
**Complications**	**Gyneco-obstetrical**	**Pediatric**	**Emergency**	**Total**
	**(N = 43)**	**(N = 36)**	**(N = 4)**	**(N = 83)**
Nausea	1 (2.3)	2 (5.6)	0 (0.0)	3 (3.6)
Vomiting	0 (0.0)	2 (5.6)	0 (0.0)	2 (2.4)
Chills	4 (9.3)	2 (5.6)	0 (0.0)	6 (7.2)
Hematuria	3 (6.9)	3 (8.3)	0 (0.0)	6 (7.2)
Anxiety	1 (2.3)	0 (0.0)	0 (0.0)	1 (1.20)
Anaphylaxis	1 (2.3)	0 (0.0)	1 (25.0)	2 (2.4)
Tachycardia	1 (2.3)	0 (0.0)	0 (0.0)	1 (1.2)
Icteria	2 (4.6)	0 (0.0)	0 (0.0)	2 (2.4)
Sudden diarrhea	2 (4.6)	0 (0.0)	0 (0.0)	2 (2.4)
Hyperthermia	0 (0.0)	0 (0.0)	1 (25.0)	1 (1.2)
Total	15 (34.9)	9 (25.0)	2 (50.0)	26 (31.3)

Data are frequencies (%) of transfusion complications recorded in each wards.

## Discussion

In this study, we recorded 477 blood donors and 83 recipients. Volunteer donors accounted for 10.5% and family donors for 89.5%. There was statistically significant difference in blood donation between volunteer donors and family donors (p = 0.03). We noticed a higher proportion of volunteer donors compared to family donors attending the LHD blood center. The reasons for increased voluntary donation within the study population can be explained by various factors, including previous loss of a relative due to lack of blood, or the quest for their HIV status. Family donors are those who donate blood to acquaintances (relatives and friends) in urgent need. Our results are similar to those reported in other studies conducted in several countries [7,18]. Contrarily to our study, some studies conducted over long periods in France and Turkey have shown that blood supply is mainly based on volunteer donation [6,19]. With the presidential decree signed on the 5^th^ April 2013, recognizing the utility of the “National Organization of volunteer blood donors, we can expect in the near future that voluntary blood donation becomes more frequent in Cameroon.

We reported a statistically significant difference between mean ages in the two donor groups (p = 0.017), with volunteer donors dominating the older age group. The number of blood donation was higher in the 23–33 years old group, which is consistent with a study conducted in 2010 at the LHD [20]. This number decreased considerably with age and volunteer donors represented the majority of donors. We can hypothesize that many persons of more than 30 years old and probably having professional activities, voluntarily make blood donation, unlike young people who may be forced to make blood donation for family reasons.

Most donors had a secondary-school education, consistent with a similar study in Côte d'Ivoire [21]. Male/female sex ratio was 4/1 for all donors in our study. It was 2.8/1 from voluntary donors and 4.1/1 from family donors. In 2010, Dikosso [20] also found a higher frequency of men among both donors groups; male/female sex ratio of 8/1 for family donors and 2/1 for volunteer donors at the HLD. Our findings are consistent with previous observations in Cameroon [7,20,22], Tanzania [18], and in a multicenter study involving seven sub-Saharan African countries [18] that showed less representation of women (< 30%) as blood donors, whether volunteer or not. This could be explained by physiological differences between men and women, who are not allowed to make blood donation during menstruation, lactation or in pregnancy.

In our donor population, men are more infected than women, but this difference was not significant. This trend has also been reported in Cameroon [20] and in Côte d’Ivoire [21]. HIV seroprevalence among blood donors was 1.77%. This prevalence is lower than that found in other localities of Cameroon (Edéa, Douala or Yaoundé) with prevalences between 4.1% and 7.9% [20,23,24]. This prevalence is also lower than the estimated national HIV prevalence of 2.6% among blood donors in 2011 [25]. The same year, the overall HIV seroprevalence in Cameroon was estimated to be 4.3% and 4.6% in the coastal region [26]. In our study, HIV infection was found only in 8/406 (~2.0%) family donors. The relative low prevalence as observed in this study could be explained by the relatively short recruitment period of 4 months compare to > 6 months in others studies. Additionally, this could result from a change in people's attitudes following the efforts of the National Committee for AIDS Control to prevent HIV transmission. Another parameter that could explain this difference is our sample size that is lower than in similar studies. HIV prevalence in our study and in all studies conducted in blood donors from 2003 was higher than that found (0.4%) in Yaoundé in the 1990 [22]. This could be explained by the introduction of 4^th^ generation tests that are more sensitive [8].

HBV infection was found in 3.5% of the donor population. It was 2.1% (1/48) among volunteer donors and 3.7% (15/413) among family donors. These prevalence values are lower than the prevalence reported in Cameroon in 2003, 2004, and 2012, that were 10.7%, 9.9% and 10.1%, respectively [23,24,27].

HCV seroprevalence in our donor population was 1.35%. It was 4.2% (2/47) among volunteer donors and 1.0% (4/396) among family donors. The overall prevalence was lower than that reported (2.3%) in 2003 by Koanga *et al.*[7] in the LHD, but identical to the 1.6% prevalence reported in Tanzania in 2006 by Mecky *et al.*[18]. This difference could result from differences in the used diagnostic tests. In the study conductyed by Mogtomo, HCV diagnosis was performed only by ELISA, whereas a combination of RDT and ELISA were employed in our studies. National HCV seroprevalence was estimated to 13.8% in 2002, with regional variations [28].

The prevalence of *T. Pallidum* infection is high in our study. The prevalence in volunteer donors was 2.1% while it was 8.88% among family donors. The overall seroprevalence among all donors was 8.1%, similar to the 2003 prevalence of 7.9% in the same hospital [7]. It was higher than the 5.7% reported in the regional hospital of Edea in 2012 [23], and lower than the 9.1% found at the University Teaching Hospital of Yaoundé in 2003 [24]. In other studies conducted in Cameroon and elsewhere [12,13], high prevalence of *T. pallidum* infection have been reported. Syphilis seems to be overlooked in Cameroon and there is no awareness campaign for this infection. Most are unaware of their *T. pallidum* status until probably at the moment of blood donation when the infection is diagnosed. Another problem is the absence of serological results given to family donors in LHD. This suggests that family donors with positive *T. pallidum* test do not know their status and could therefore continue to serve as reservoirs for the bacteria, which can lead to infertility. Taken together, these findings indicate that bacterial contamination is the major risk of infection during blood transfusion. In addition, we report a single case of co-infection HCV/*T. pallidum* in the study population. In a study conducted in China, HIV/*T. pallidum* and HBV/*T. pallidum* co-infections were noticed in addition to HCV/*T. pallidum* infected cases [13]. The prevalence of *T. pallidum* infections and co-infections was probably underestimated in our study given that the HIV positive participants were not screened for *T. pallidum* infection.

None of the assessed risk factors was significantly associated with infection in our study. This is consistent with the study conducted in Australia between 2005 and 2010 in a population of infected blood donors [5]. However, Kra *et al.*, in 2001 had found that having multiple partners, unprotected sex, or history of hazardous behavior (injections, blood transfusions) were risk factors for positive HBsAg among blood donors in Côte d'Ivoire [21].

We recorded 83 blood recipients (male/female sex ratio of 3.6/1) in the Pediatric, Gyneco-obstetrical and emergency wards. These wards were chosen because they presented the highest blood requests as noted in blood bank records. The high number of female recipient is due to the high frequency of anemia in Gyneco-obstetrical ward. Various complications have been identified from medical records of patients during and after transfusion, and 31.32% of recipients had at least one complication. This value is lower than that obtained by Mbanya *et al*. (> 50%) between 1994 and 1998 in Cameroon [11]. Complications were mainly found in Gyneco-obstetrical ward, as well as the number of blood transfusions. This could be explained by frequent bleeding in women during childbirth. Studies have shown that transfusion occurs when blood loss exceeds 500 ml [29,30]. Chills and hematuria were the most frequent complications (14.5% of recipients) and accounted for 46.1% of all complications. Mbanya *et al.* reported febrile reactions and urticaria in 40.1% and 19.4% recipients respectively [11]. Chills and hematuria are signs and symptoms of acute or delayed hemolytic reactions. Chills may be due to the fact that some transfused patients did not receive blood of the same group. Due to lack of blood, several patients received O group blood although not belonging to this group. The O group was predominant among all blood donors in our study (52.2%), and blood transfusion in this group was generally for emergency purposes. A study conducted by Mandengue *et al*. (2003) in the LHD showed the existence of several blood systems and also, that the distribution among donors and recipients is not always the same [31]. Hematuria due to non-immune hemolysis of red blood cells compatibility was particularly observed in Sickle Cell Disease (SCD) in our study. Among the recipients, 10 SCD patients agreed to participate in this study. SCD patients are generally subject to blood transfusions. A study conducted in Yaoundé in 2012 by Ngo Sack *et al*. showed that SCD patients may receive more than 10 transfusions and that the risk of infection increases with the number of transfusion [32]. Another study conducted in Libreville in 2002 by Moussaoui *et al*. showed the benefits of these transfusions used in systematic prophylaxis [33].

Among the recipients, 57 (68.7%) were transfused for the first time and 26 (31.3%) had received previous transfusions. The number of previous transfusion was higher in Gyneco-obstetrical and Pediatric wards. In the Pediatric ward, anemia due to malaria was the main indication for transfusion, followed by sickle cell vaso-occlusive crisis. In a study conducted in Benin, malarial anemia was the main indication for blood transfusion in Pediatric wards, followed by malnutrition, viral and bacterial infections (pneumonia, meningitis, measles, typhoid fever), and SCD [34]. Our results, however, do not reflect the morbidity associated with sickle cell disease.

### Limitations and strengths

The weaknesses of this study include: 1) the small sample size (83 recipients) and the lack of funding which limited our ability to explore seroconversion of viral and bacterial infections in blood recipients and 2) the low representation of all sub-regional hospitals in this study that potentially could impose significant bias in the obtained data; for instance, if a patient visited another hospital with an acute complication, our institution database may not have a record of that visit.

Compared to previous reports in this area, our study is one of the few researches to investigate transfusion-related complications in blood recipients attending the HLD, while past research has largely focused on blood donors. This study provides a comprehensive analysis of the prevalence of both viral and bacterial infections among blood donors in Douala, providing additional insight into TTI-associated disease burdens and opportunities for prevention from a local standpoint.

## Conclusion

The aim of this study was to investigate the prevalence of TTIs among blood donors, and complications due to transfusion in blood recipients attending the LHD. HIV, HBV, HCV and *T. pallidum* infections in blood donors were recorded with a high proportion of *T. pallidum* infection, representing over 53.84% of all infections. Infection prevalences were two folds higher in family donors than in volunteer donors, suggesting a low-risk in this later group. We also reported hematuria in the majority of complications found in blood recipients. However, studies on malaria infection and on seroconversion rate of viral or bacterial infections in blood recipients are also required. Taken together, our findings will contribute to significantly to ongoing efforts to improve transfusion-related haemovigilance in blood recipients at the HLD and the sub-region.

## Competing interests

The authors declare no conflicts of interest.

## Authors’ contributions

CEEM conceived and designed the study. EGES collected the data. CEEM, FNS, MM and LGL coordinated the study. Data analysis and interpretation: CEEM and EGES. The manuscript was drafted by CEEM and all authors contributed to the revision and approved the final manuscript.

## Pre-publication history

The pre-publication history for this paper can be accessed here:

http://www.biomedcentral.com/2052-1839/14/5/prepub
